# Primary Cardiac Tumor in the Left Atrium: A Diagnostic Challenge

**DOI:** 10.7759/cureus.39443

**Published:** 2023-05-24

**Authors:** Helena Rodrigues, Ana Rita Ramalho, Isabel Correia, Rogério Ferreira, Lèlita Santos

**Affiliations:** 1 Internal Medicine Department, Centro Hospitalar e Universitário de Coimbra, Coimbra, PRT

**Keywords:** cardiac tamponade, pericardial effusion, heart failure hospitalization, constitutional symptoms, cardiac mass tumor, primary cardiac tumor

## Abstract

An elderly man presented to the emergency department with shortness of breath, peripheral edema, and significant weight loss. Blood tests revealed anemia and elevated inflammatory markers, and chest imaging showed a massive left pleural effusion. During hospitalization, he developed subacute cardiac tamponade, and pericardiocentesis was performed. Further imaging revealed a primary malignant cardiac tumor with extensive infiltration of the cardiac tissue, and biopsy was deemed impossible due to the tumor’s location. The most likely diagnosis was angiosarcoma. The cardiac surgery team evaluated the case and considered it inoperable due to the tumor’s extensive infiltration. The patient is currently under the regular care of a palliative care team. This case underscores the difficulties of diagnosing primary cardiac tumors, particularly in elderly patients with comorbidities. Despite advances in imaging and surgical techniques, the prognosis for malignant cardiac tumors remains poor.

## Introduction

Cardiac masses are extremely rare, with a reported incidence ranging from 0.001% to 0.3% in various studies [1]. They can be further classified as benign tumors, malignant tumors (primary and secondary), and tumor-like conditions [2,3]. They are commonly diagnosed in children and young adults and are often asymptomatic until complications develop. Cardiac tumors may present with variable symptoms depending on their size, location, and structures involved. Symptoms often relate to the structures affected and can cause cardiac, embolic, and systemic symptoms [2,4,5]. Due to their nonspecific presentation, diagnosis and management are often delayed [6]. In the elderly population, diagnosing primary cardiac tumors is even more challenging due to the presence of other comorbidities that can mask or mimic symptoms; therefore, the diagnosis of a cardiac tumor requires a high degree of suspicion. Echocardiography is the first-line imaging technique, which in most cases allows to assess the tumor size, location, and attachment site and its hemodynamic effect [2-4]. However, the best imaging technique is cardiac magnetic resonance, as it allows to characterize the mass and assess the function of the cardiovascular system [3,7]. Nevertheless, a definitive diagnosis is only possible with biopsy and histopathological evaluation [2,6]. Treatment and outcome depend on tumor characteristics, location, metastatic involvement, and comorbidities [3,5]. Despite the advances in imaging and surgical techniques, the prognosis for malignant cardiac tumors remains poor, with a median survival of approximately seven months for primary cardiac sarcoma [8].

We present a case of a primary cardiac tumor in an elderly man that highlights the challenges in diagnosis of this entity.

This case was presented as a poster at the Portuguese Congress of Internal Medicine on May 5, 2023.

## Case presentation

A man in his mid-80s presented to the emergency department with complaints of shortness of breath at rest, orthopnea, paroxysmal nocturnal dyspnea, and peripheral edema that had progressively worsened over the past four days. He also complained of anorexia, weight loss of 15 kg in the last year, generalized myalgia, and strength loss.

On physical examination, his vital parameters were normal. He was polypneic, had diminished breath sounds on the left lung base, and had pretibial edema.

He had a pacemaker and a past medical history of atrial fibrillation, non-stratified heart failure, diabetes, hypertension, hyperlipidemia, anemia, and prostate cancer in remission. The patient’s usual medication included edoxaban 60 mg, furosemide 40 mg, perindopril 5 mg, amlodipine 5 mg, simvastatin 40 mg, pantoprazole 40 mg, folic acid 5 mg, ferrous sulfate 329.7 mg, metformin 700 mg, sucralfate 1000 mg, budesonide/formoterol 160/4.5 ug, and quetiapine 25 mg.

Blood tests revealed a hemoglobin level of 7.2 g/dL and elevated inflammatory parameters, including a white cell count of 13.4 × 109/L and a C-reactive protein level of 7.34 g/dL (Table 1). A chest radiography showed cardiomegaly and a massive left pleural effusion, and an electrocardiogram showed a pacemaker rhythm.

**Table 1:** Blood test results during admission NT-proBNP, N-terminal prohormone of brain natriuretic peptide

Blood test	Admission day	During decompensation	At discharge	Reference values
Hemoglobin (g/dL)	7.2	9.2	8.6	11.8-15.8
Hematocrit (%)	23	28.3	27	37-49
White blood cell count (10^9^/L)	10.6	15.8	11.6	3.6-10.5
Neutrophil count (10^9^/L)	8.35	13.94	9.42	1.5-7.7
C-reactive protein (mg/dL)	7.18	8.51	10.49	<0.5
Creatinine (mg/dL)	0.94	2.42	1.43	0.72-1.18
Sodium (mmol/L)	135	130	138	136-146
Potassium (mmol/L)	4.1	5.2	3.8	3.5-5.1
Aspartate aminotransferase (U/L)	13	32	12	<35
Alanine aminotransferase (U/L)	9	11	8	<45
Alkaline phosphatase (U/L)	159	167	170	30-120
γ-Glutamyl transpeptidase (U/L)	45	46	39	<55
NT-proBNP (pg/mL)	2396	7612	Not measured	<450

The patient was hospitalized for non-stratified decompensated heart failure, worsening anemia, and massive left pleural effusion

During hospitalization, the patient experienced a sudden worsening of his clinical condition, which is associated with the development of subacute cardiac tamponade. Pericardiocentesis was performed, which was the draining of 1600 mL of hematic fluid. Biochemical analysis of the fluid revealed low glucose levels (45 mg/dL) and high levels of protein (7.2 g/dL) and LDH (532 U/L). Cytological examination of the fluid revealed no malignant cells, and cultures were negative. To determine the cause of the subacute pericardial effusion, various exams were performed, including serologies and cultures, which were negative. Autoimmunity tests were also negative, and metabolic, radiation, and drug causes were excluded. The most likely cause at this point was neoplastic. 

**Figure 1:**
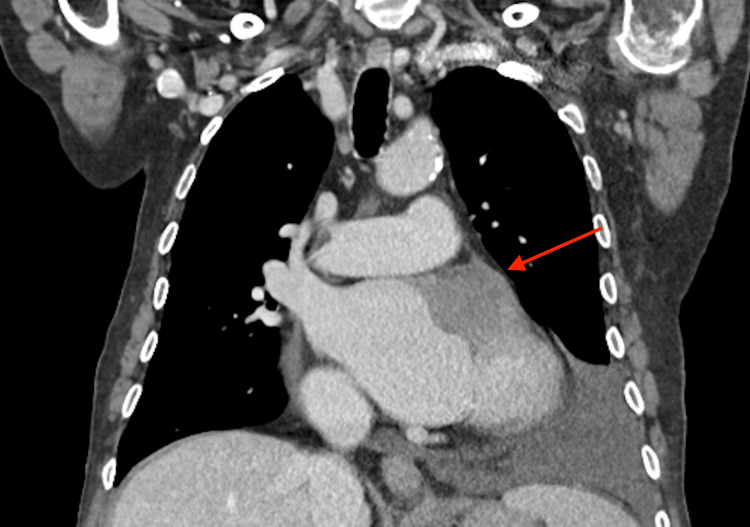
Coronal section of CT-thorax demonstrating hypodensity at the level of the left auricle: left pleural effusion

**Figure 2:**
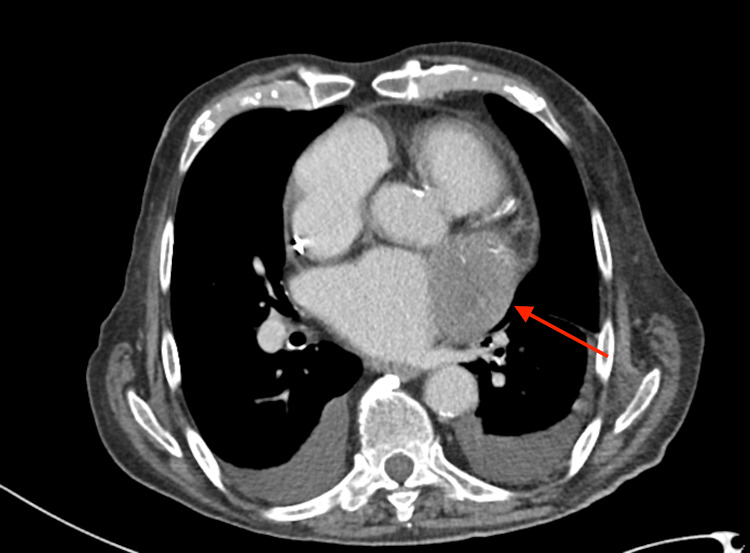
Axial section of CT-thorax demonstrating hypodensity at the level of the left auricle

Computed tomography of the chest, abdomen, and pelvis revealed a mass in the left auricula (Figures 1, 2), but no extracardiac malignancy was identified, increasing suspicion of a primary cardiac malignant tumor.

**Figure 3:**
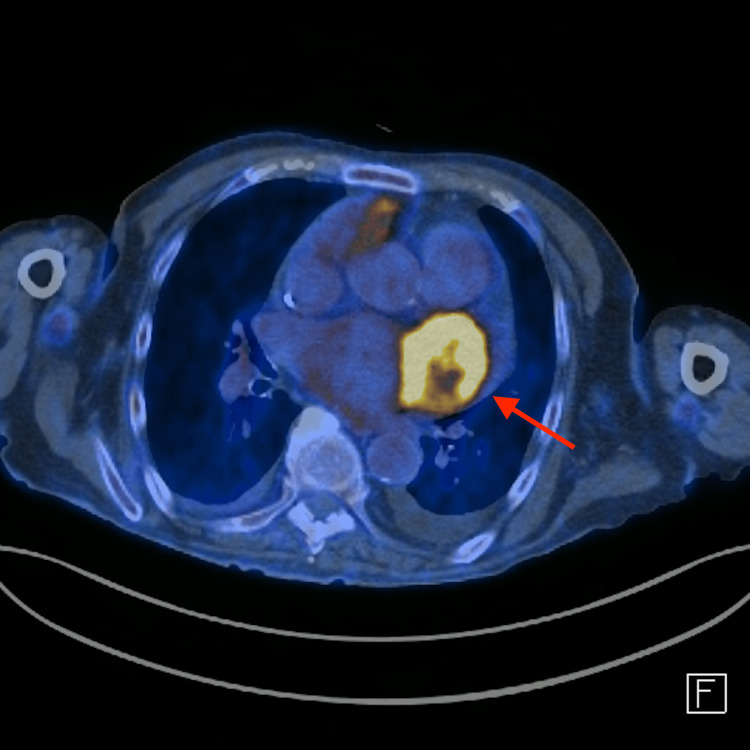
PET-FDG scan revealed a voluminous hypermetabolic mass in the heart, seemingly originating from the lateral aspect of the left auricle The mass measured approximately 56 × 48 mm in its largest axial axes, with a maximum standardized uptake value (SUVmax) of 14.3. The mass exhibited anatomical and functional characteristics consistent with a malignant neoplastic mass with a high metabolic rate.

**Figure 4:**
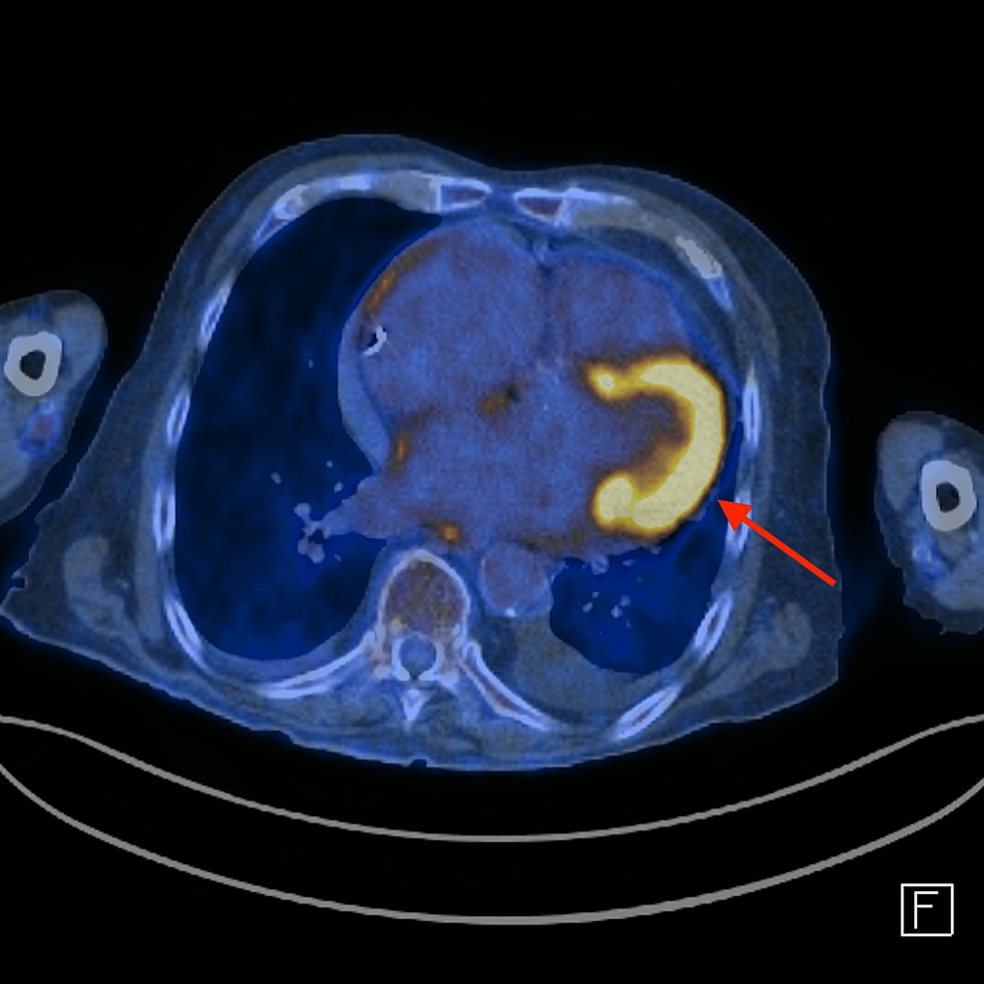
PET scan revealed a mass that appeared to have no clear separation between it and the latero-basal region of the left ventricle, and its limits were ill-defined

Transesophageal echocardiography revealed the presence of a heterogeneous vascularized mass with irregular borders in the anterolateral wall of the left atrium and with infiltration of the external region of the mitral valve and lateral wall of the left ventricle. Due to the location and extent of the tumor, a biopsy for diagnosis was deemed impossible, and a presumptive diagnosis of a primary cardiac tumor was made. PET-FDG confirmed the suspicion of a high metabolic grade malignant lesion (Figures 3, 4).

Based on the location and characteristics of the tumor, the most probable diagnosis was an angiosarcoma, which is the most common primary malignant tumor of the heart, known for its widespread local invasion and potential to cause pericardial effusion [4,5]. Rhabdomyosarcoma is the second most common primary malignant tumor, but its most common location is in the ventricles and is more frequent in children and adolescents [4,9]. Another possibility is undifferentiated pleomorphic sarcoma, which occurs more commonly in the left atrium [4].

Although rare, cardiac metastasis from prostate cancer should be considered in this patient due to his medical history. However, a PET scan has excluded this possibility. The PET results also excluded a benign tumor, so myxoma, which commonly occurs in the left atrium (90% of cases) [9], was less likely.

The cardiac surgery team evaluated this case and deemed it inoperable due to extensive infiltration of the cardiac tissue and perioperative risk. Due to the patient’s age, cardiac transplantation or open chest surgery for biopsy was not considered a viable option. As a result, chemotherapy could not be initiated without a histopathologic diagnosis.

The patient is currently under observation, and the best medical approach for his comorbidities has been established. The patient is being closely monitored by a palliative support team.

## Discussion

This case report describes a primary cardiac tumor in an elderly man and highlights the challenges in diagnosing and managing this condition.

As noted earlier, primary cardiac tumors are extremely rare [1] and have a nonspecific presentation. The symptoms presented by this patient were easily confused with decompensated heart failure due to anemia, and it was the development of a subacute cardiac tamponade that raised higher suspicion of an alternate diagnosis. During pericardiocentesis, echocardiography did not identify a cardiac mass. However, the drained hematic fluid guided us to explore differential diagnoses. A cardiac magnetic resonance imaging would have been an excellent imaging technique to evaluate the mass’s topography, morphology, size, vascularization, composition, and potential hemodynamic effect [2,7]. Unfortunately, it was contraindicated because the patient had a pacemaker. The PET scan was important to evaluate the metabolic activity of the tumor, to exclude secondary malignancy, and for staging [2].

Even though only a biopsy and histopathological evaluation would allow a definitive diagnosis [5], due to the patient status, comorbidities, and tumor location and extension, open chest surgery for biopsy and surgical resection of the tumor were deemed impossible. As described in a retrospective study by Isogai et al. [10], elderly patients with lower Barthel Index are less likely to undergo surgery. In these cases, treatment is often directed with palliative care.

The prognosis of primary malignant tumors remains poor despite the technological advancements due to their aggressiveness, high rates of relapse, limited treatment options available (particularly for inoperable cases), and possible complications [5].

## Conclusions

Primary cardiac tumors are extremely rare, and they may manifest with symptoms that are difficult to pinpoint, causing a delay in diagnosis and an increased burden of illness. Detecting them necessitates a high level of suspicion, and imaging techniques such as echocardiography and cardiac magnetic resonance can assist with the diagnosis. Nonetheless, a biopsy and histopathological evaluation are required for a definitive diagnosis. Treatment and prognosis are determined by the type and location of the tumor. Treatment options may be limited in elderly patients with comorbidities who have an inoperable tumor. In certain instances, close observation and palliative support may be the most appropriate course of action.

## References

[1] Reynen K (1996). Frequency of primary tumors of the heart. Am J Cardiol.

[2] Tyebally S, Chen D, Bhattacharyya S (2020). Cardiac tumors: JACC CardioOncology state-of-the-art review. JACC CardioOncol.

[3] Poterucha TJ, Kochav J, O'Connor DS, Rosner GF (2019). Cardiac tumors: clinical presentation, diagnosis, and management. Curr Treat Options Oncol.

[4] Campisi A, Ciarrocchi AP, Asadi N, Dell'Amore A (2022). Primary and secondary cardiac tumors: clinical presentation, diagnosis, surgical treatment, and results. Gen Thorac Cardiovasc Surg.

[5] Joshi M, Kumar S, Noshirwani A, Harky A (2020). The current management of cardiac tumours: a comprehensive literature review. Braz J Cardiovasc Surg.

[6] Strecker T, Rösch J, Weyand M, Agaimy A (2012). Primary and metastatic cardiac tumors: imaging characteristics, surgical treatment, and histopathological spectrum: a 10-year-experience at a German heart center. Cardiovasc Pathol.

[7] Esposito A, de Cobelli F, Ironi G (2014). CMR in assessment of cardiac masses: primary benign tumors. J Am Coll Cardiol Img.

[8] Yin K, Luo R, Wei Y (2021). Survival outcomes in patients with primary cardiac sarcoma in the United States. J Thorac Cardiovasc Surg.

[9] Maraj S, Pressman GS, Figueredo VM (2009). Primary cardiac tumors. Int J Cardiol.

[10] Isogai T, Yasunaga H, Matsui H, Tanaka H, Hisagi M, Fushimi K (2017). Factors affecting in-hospital mortality and likelihood of undergoing surgical resection in patients with primary cardiac tumors. J Cardiol.

